# Errors in the arterial blood pressure measurement

**DOI:** 10.1186/cc10819

**Published:** 2012-03-20

**Authors:** F Franchi, V De Palo, A Faltoni, S Cecchini, L Cubattoli, P Giomarelli

**Affiliations:** 1 University of Siena, Italy; 2 Hospital of Siena, Italy

## Introduction

The artefacts affecting arterial wave morphology may compromise recorded values of arterial blood pressure (ABP) and can lead to therapeutic errors. The aim of this study is to evaluate the errors between invasive and noninvasive arterial pressure values, the incidence of artefacts due to an inadequate dynamic response of the transducer-tubing system, and their detection by the ICU staff.

## Methods

Seventy-five consecutive patients (50 male, mean age 55 ± 18) admitted to the ICU for heterogeneous pathologies were enrolled. Inclusion criteria were: the presence of an intra-arterial catheter (IAC) for invasive blood pressure monitoring, and age >18 years. Pregnancy was excluded. At admission and every time the IAC was replaced we acquired invasive systolic, diastolic, and medium arterial pressure values (I-SP, I-DP, I-MP) during hemodynamic stability (variations of mean arterial pressure <10%); at the same time, noninvasive systolic and diastolic arterial pressure values (Ni-SP, Ni-DP) were measured with a sphygmomanometer at the same arm of the IAC. Noninvasive medium arterial pressure (Ni-MP) was calculated as follows: (SP + 2DP)/3. At every time of the study, before ABP value acquisition, medical and nursing staff answered a questionnaire on the reliability of the arterial waveform. The staff could perform the fast flush test if considered appropriate. However, the fast flush test was executed by the main investigator at the end of questionnaire in all patients. Bland-Altman analysis was performed.

## Results

We compared 130 pairs of Ni-SP, Ni-DP and Ni-MP and I-SP, I-DP and I-MP. The mean bias between Ni-SP and I-SP was -11 mmHg (limit of agreement (LoA) -43.6 to 21.4 mmHg). The mean bias between Ni-DP and I-DP and between Ni-MP and I-MP was 6.1 mmHg (LoA -15.5 to 27.7 mmHg) and 0.37 mmHg (LoA -21.0 to 21.7 mmHg), respectively. We performed the fast flush test 130 times; an inadequate dynamic response of the transducer-tubing system was observed 55 times: in 45 cases the arterial signal was underdumped and in 10 cases was overdumped. The arterial dumping was correctly detected by the medical staff in 95% of cases, by nursing staff and postgraduates in 35% of cases.

## Conclusion

The bias between invasive and noninvasive ABP measure can be relevant and mislead in the therapeutic management. These errors can be avoided by identifying the artefacts that affect arterial signal and so the ICU staff must pay attention to the recognition of arterial dumping in critically ill patients.
